# Diminished Health Returns of Educational Attainment Among Immigrant Adults in the United States

**DOI:** 10.3389/fpsyt.2020.535624

**Published:** 2020-11-27

**Authors:** Shervin Assari, Sharon Cobb, Adolfo G. Cuevas, Mohsen Bazargan

**Affiliations:** ^1^Department of Family Medicine, Charles R. Drew University of Medicine and Science, Los Angeles, CA, United States; ^2^Department of Urban Public Health, Charles R. Drew University of Medicine and Science, Los Angeles, CA, United States; ^3^School of Nursing, Charles R. Drew University of Medicine and Science, Los Angeles, CA, United States; ^4^Department of Community Health, Tufts University, Boston, MA, United States

**Keywords:** population groups, immigration, nativity, socioeconomic status, socioeconomic position, psychological distress, self-rated health, chronic disease

## Abstract

**Objectives:** Marginalization-related diminished returns (MDRs) refer to weaker health effects of educational attainment for socially marginalized groups compared to the socially privileged groups. Most of the existing literature on MDRs, however, has focused on marginalization due to race, ethnicity, and sexual orientation. Thus, very limited information exists on MDRs of educational attainment among immigrant populations in the United States.

**Aims:** Building on the MDRs framework and using a nationally representative sample of US adults, we compared immigrant and native-born adults for the effects of educational attainment on psychological distress, self-rated health (SRH), and chronic diseases (CDs).

**Methods:** The 2015 National Health Interview Survey (NHIS) has enrolled 33,672 individuals who were either immigrant (*n* = 6,225; 18.5%) or native born (*n* = 27,429; 81.5%). The independent variable (IV) was educational attainment, which was treated as a categorical variable. The dependent variables included psychological distress, SRH, and CDs, all of which were dichotomous variables. Age, gender, race, ethnicity, and region were confounders. Immigration (nativity status) was the moderator.

**Results:** Higher educational attainment was associated with lower odds of psychological distress, poor SRH, and CDs. However, immigration showed a significant statistical interaction with college graduation on all outcomes, which were suggestive of smaller protective effects of college graduation on psychological distress, poor SRH, and CDs for immigrant than native-born adults.

**Conclusions:** In the US, the associations between educational attainment and psychological distress, SRH, and CDs are all weaker for immigrant than native-born adults. To prevent health disparities, it is essential to decompose health inequalities that are due to low educational attainment from those that are due to diminished returns of educational attainment (i.e., MDRs). There is a need to help highly educated immigrant adults secure positive health outcomes, similar to their native-born counterparts. Such changes may require bold and innovative economic, public, and social policies that help immigrant adults to more effectively mobilize their educational attainment to secure tangible outcomes. Elimination of health disparities in the US requires efforts that go beyond equalizing access to education.

## Background

Extensive theoretical and empirical work has established a connection between socioeconomic status (SES) indicators, particularly educational attainment and a wide range of physical and mental health outcomes (1–3). For example, individuals with high levels of formal education are less likely to report depression (4), anxiety (5), suicidal ideas (6), or psychological distress (7, 8), and are more likely to report happiness (9). Various types of social marginalization (e.g., based on race, ethnicity, sexual orientation, and immigrant status) may reduce the salience of educational attainment on the mental well-being of populations (10–13). Immigration also exposes populations to a wide range of stressors and social risk factors that increase risk of mental distress (14–17).

Research on intersectionality (18–20) has well-established that subpopulations, however, differ widely in the health effects of educational attainment (21–24) and other SES indicators (25–27). According to the marginalization-related diminished returns (MDRs) (28, 29), the effects of educational attainment (30) on mental (31, 32) and physical health (33–36) are weaker for racial and ethnic minority adults than the majority group. Although comparisons of Black and White people (22, 23) shape most of this literature, some similar results have been reported for Hispanics (37, 38), Asian Americans (39), Native Americans (40), and even members of the lesbian, gay, bisexual, and transgender (LGBT) community (41–43). For example, educational attainment shows weaker effects on mental well-being (41), smoking (42), and obesity (43) for LGBT adults than non-LGBT people.

While there is compelling evidence that racial, ethnic, and sexual minority adults tend to gain fewer health benefits from their educational attainment (33), it is yet unknown if the same MDRs may also be relevant to the comparison of immigrant and native-born people. Similar to Black (31), Hispanic (37, 44), and LGBT (41–43) individuals, immigrant adults experience marginalization and discrimination by the host society (45–49). Exposure to unequal treatment, in turn, reduces immigrant adults' participation in social activities and labor market. Societal and structural factors such as social stratification, residential segregation, labor market discrimination, and low quality of education in urban areas may hinder the full benefit of education. Due to social and economic adversities, educational attainment may not be an equalizer of health for marginalized groups. Nevertheless, little is known on whether the association between education and health differs by nativity status.

Previous research on immigrant populations has shown considerable differences in health status between immigrant and native-born individuals. A pattern known as the healthy immigrant effect (50–52) suggests that despite experiencing a wide range of social disadvantages (e.g., lower levels of education and income, poor access to healthcare), immigrant individuals show better health profile than native-born individuals. For example, a large body of research has established lower mortality risk of Latino immigrant adults compared to White adults (53–56). For Hispanics, this phenomenon has been coined the Hispanic or Latino mortality paradox because Hispanic and Latino immigrants have lower education and higher exposure to social risk factors such as economic adversities and social disorder, yet have lower mortality risk than White adults (50, 54, 57–65). The relationship between nativity status and health cannot be interpreted unequivocally, as immigration-related factors, such as country of origin, reason behind immigration, and age play important roles in the relationship (15, 16, 66). For instance, the degree to which immigrant adults acculturate and adopt the host country's culture influences their health outcomes, health behaviors, and well-being (67–71).

Marginalization and discrimination are major drivers of immigrant peoples' poor health (46, 47, 49, 72–74). Another factor is that they may live in ethnic enclaves with low access to resources (75–80). Another factor is that some immigrant groups have high and some have low SES (81–85). For example, in the US, Asian Indian, Cuban, Asian American, and Chinese people have higher SES while Mexican, Puerto Rican, and Filipino people have lower SES compared to White people in the US. These are a source of heterogeneities and should be considered in future analysis of the association between immigration and health (81–85).

Despite immigrant populations showing better health profiles than native-born people, they may not gain the same health benefits provided by social structures. Xenophobia can be seen as another source of poor health and well-being of immigrant populations (86, 87). Exposure to Xenophobia and related hate and discrimination, may, however, not be the same across regions (88). As such, experiences of immigrant people may widely depend on a wide range of geopolitical factors such as which political party that holds administrative and legislative power (89). Even within the same region, drastic changes may occur as a result of elections and changes in the leadership of countries, states, and even cities (90). Life condition of immigrant populations and resources that they can access can be heavily influenced by the political party that takes power (91). In the US, for example (92), a major part of the nationalist, populist political rallies have focused on generating fear in the voters. These policies commonly focus on safety, crime, and scarcity of jobs and resources so immigrant individuals are perceived as a security threat and competitors to jobs (91). Such policies and rhetoric's have historically helped the conservative politicians to collect votes (86). As such, there has been an inertia against advocating for the health and well-being of immigrant adults by the right-wing policymakers. Two latest examples of these changes are remarks by Donald Trump in the US (91–93), and Boris Johnson and Nigel Farage in the UK (94). As a result, many undocumented immigrant people, and even legal immigrant adults, may face fear, hate crimes, and discrimination (95).

Intersectionality research has been also used to study the health status of immigrant populations in the US and other countries (96). Another source of health inequalities in immigrant populations is low SES, which may partially explain poor health of some of the groups of immigrant adults, compared to the native people (97, 98). Not all groups of immigrant populations, however, have lower SES than the native-born people. In the US, for example, Cubans, Asian Americans, and Asian Indians have comparable or even higher SES than the average for Whites (99). There is also some research on this topic, showing that SES may generate less outcomes for immigrant than native-born individuals (100). Thus, all this literature and all these patterns suggest that health of immigrant populations have major nuances and complexities, and one size does not fit all (50, 59, 60, 62, 101, 102). These nuances emerge as a result of complex interactions between immigration, acculturation, culture, and visible and invisible identity markers that depend on country of origin and SES and clash with various systems of privilege in the host county (103, 104). Intersectionality framework has helped us understand some of these complexities (105).

## Aim

By borrowing data from the National Health Interview Survey (NHIS), and informed by the MDRs and intersectionality frameworks, we conducted a cross-sectional study to compare immigrant and native-born people in the US for the association between educational attainment and income and psychological distress of American adults. As suggested by both intersectionality and MDRs, we conceptualized immigration status as a combination of visible or invisible marginalizing identities that would reduce access of individuals to the opportunity structure, deny them their dignity and privilege, and expose them to prejudice and injustice. Thus, we expected diminished effects of educational attainment on psychological distress, self-rated health (SRH), and chronic diseases (CDs) of immigrant compared to native-born people. While the same research question could be asked in an international scope, the focus of this paper is exclusively the US.

## Methods

Data of the NHIS 2015 were used. The NHIS is the primary source of information regarding the physical health status of American adults 18 years or older. The NHIS sample is composed of US residents, civilians, and non-institutionalized people.

## Participants and Sampling

The NHIS used a multistage sampling: First, was to sample 428 primary sampling units (PSUs) drawn from 1,900 geographically defined PSUs. All 50 US states and the District of Columbia had PSUs in the study. The PSUs were either a metropolitan statistical area, a single county, or a small group of contiguous counties.

## Process

The data are collected by the National Center for Health Statistics (NCHS), which is a part of the Centers for Disease Control and Prevention (CDC). The U.S. Census Bureau collects the data. Data are collected via face-to-face interviews in participants' households. On some occasions, this face-to-face interview is followed or replaced by telephone interview.

## Participants

The total sample in this study was 33,672 individuals who were either immigrants (*n* = 6,225; 18.5%) or native-born (*n* = 27,429; 81.5%). We did not impose any exclusion criteria.

## Measures

### Predictor

#### Educational Attainment (EA)

Educational attainment was operationalized as a categorical variable with four levels. Participants were asked about the number of years of schooling. The educational levels included (1) <12 years (reference group), (2) 12 years, (3) some college education, and (4) college graduate.

### Moderator

#### Immigration Status

Nativity was self-reported. All participants were asked if they were born in the US. The responses were coded 1 for immigrant and 0 for native born.

### Covariates

#### Demographic Factors

Demographic factors included age, gender, census region, race, and ethnicity. Age (years) was a continuous variable. Gender was a dichotomous measure (female = 0 and male = 1). The region was either Northeast, Midwest, South, or West. Participants self-identified their race and ethnicity. Race and ethnicity were both operationalized as categorical variables. Race included White only (reference category), Black/African American only, Native American/Alaska Native only, Asian only, Multiple race, and race group not releasable (masked or missing). Ethnicity included Non-Hispanics = 0 (reference category) and Hispanics = 1.

### Dependent Variable

#### Psychological Distress

The following items were used to measure psychological distress. (1) How often you felt so sad nothing cheers you up during the past 30 days, (2) How often you felt nervous during the past 30 days, (3) How often you felt restless/fidgety, during the past 30 days, (4) How often you felt hopeless during the past 30 days, (5) How often you felt everything was an effort during the past 30 days, and (6) How often you felt worthless during the past 30 days. Responses to these items included (0) none of the time, (1) a little of the time, (2) some of the time, (3) most of the time, and (4) all of the time. We calculated a sum score, ranging from 0 to 24, with a higher score indicating higher psychological distress.

#### Self-Rated Health (SRH)

We used the conventional single item of SRH to measure overall health. Responses were excellent, very good, good, fair, and poor. Responses were dichotomized so fair and poor reflected poor, and other responses reflected good health. Poor SRH is predictive of morbidity and mortality in clinical as well as community settings (106).

#### Chronic Diseases (CDs)

Participants were asked if they had any of the following conditions: cancer, epilepsy/seizure, sinusitis, hay fever, emphysema COPD, chronic bronchitis, weak/failing kidneys, liver condition, arthritis, carpal tunnel syndrome, hypertension, high cholesterol, diabetes, coronary heart disease, angina pectoris, stroke asthma, ulcer, and/or Crohn's disease/ulcerative colitis. The exact items read as: “Ever been told you have diabetes,” etc. We calculated a dichotomous variable that included presence or absence of any CDs, regardless of their type.

### Statistical Analyses

Given the NHIS's multistage sampling design, we needed to apply SPSS 23.0 (IBM Inc, NY, USA) for our data analysis. Using SPSS, we adjusted for the NHIS survey weights that were due to the design variables (strata, clusters, and non-response). Taylor series linearization was applied for the re-estimation of the standard errors (SEs). For descriptive statistics, we used weighted means and frequencies.

For our multivariable analyses, given our binary outcomes, we applied two logistic regression models for each outcome. In these models, either psychological distress, SRH, or CD were the dependent variable. In all these models, which were all significant, educational attainment was the independent variable, while demographic factors, income, and region were the control variables, and immigration status was the moderator. Both our models were calculated in the pooled sample that included both immigrant and native-born adults. *Model 1* did not include immigration by educational attainment interaction terms. *Model 2*, however, included immigration by educational attainment interaction terms. To generate an interaction term, three interaction terms were entered to the models: 12 years × immigrant, 13–15 years × immigrant, and 16 + years × immigrant. If these interactions were significant and positive, they would suggest that the protective effect of 12 years education, in comparison to the reference group (<12 years of education) is smaller for the immigrant than native-born groups. This would be in support of our MDRs hypothesis. For our immigrant variable, immigrants were coded as 1 and native-born coded as 0. Similarly, for race, White only was the reference group so the OR would mean the effect of a particular race relative to Whites. Similarly, non-Hispanic was the reference group, thus the effect of ethnicity referred to the effect of being Hispanic compared to non-Hispanic. Adjusted odds ratio (AOR), 95% confidence intervals (CI), SE, and *p*-values were reported. A *p* < 0.05 was considered significant.

## Results

### Descriptive Statistics

The total sample in this study was 33,672 immigrant and native-born American adults 18+ years old. Of the participants, 18.5% were immigrants, and 81.5% were native born. From the total sample, 77% were White, and 23% were a member of other racial groups. Additionally, 17% were Hispanic, and 83% were non-Hispanic. Table 1 depicts the descriptive statistics of the participants overall and based on the nativity.

**Table 1:** Descriptive statistics overall (33,672).

	**All**		**Native born**		**Immigrant**	
	***n***	**%**	***n***	**%**	***n***	**%**
Immigration status						
Native born	27,429	81.5	27,429	100.0	**–**	**–**
Immigrant	6,225	18.5	**–**	**–**	6,225	100.0
Race^*^						
White only	25,831	76.7	21,937	80.0	3,881	62.3
Black only	4,673	13.9	4,033	14.7	637	10.2
Native American/Alaska Native only	392	1.2	311	1.1	81	1.3
Asian only	1,983	5.9	492	1.8	1,489	23.9
Multiple race	699	2.1	609	2.2	90	1.4
Race group not releasable	94	0.3	47	0.2	47	0.8
Ethnicity^*^						
Non-Hispanics	28,080	83.4	25,128	91.6	2,942	47.3
Hispanics	5,591	16.6	2,301	8.4	3,283	52.7
Region^*^						
Northeast	4,681	13.9	4,346	15.8	1,232	19.8
Midwest	8,359	24.8	6,395	23.3	705	11.3
South	9,047	26.9	9,579	34.9	2,061	33.1
West	11,436	34.0	7,109	25.9	2,227	35.8
Gender						
Female	18,601	55.2	15,152	55.2	3,440	55.3
Male	15,071	44.8	12,277	44.8	2,785	44.7
Education^*^						
Less than 12 years	5,580	16.6	2,852	10.4	1,826	29.3
12 years	7,102	21.1	7,045	25.7	1,313	21.1
13–15 years	11,646	34.6	7,990	29.1	1,057	17.0
16+ years	9,344	27.8	9,457	34.5	1,970	31.6
Psychological distress						
No	25,266	78.3	20,512	74.8	4,741	76.2
Yes	6,990	21.7	5,792	21.1	1,196	19.2
Self-rated health (SRH) (Poor)						
Yes	28,614	85.0	23,272	84.8	5,328	85.6
No	5,046	15.0	4,147	15.1	896	14.4
Chronic diseases (CDs)^*^						
No	16,653	50.6	13,066	47.6	3,574	57.4
Yes	16,227	49.4	13,727	50.0	2,495	40.1
		**SD**	**Mean**	**SD**	**Mean**	**SD**
Age (years)^*^	49.94	18.38	50.57	18.68	47.19	16.71

* *p < 0.05 for comparison of immigrant and native born*.

Table 1 also compares the two groups. Compared to native-born people, immigrant adults were more likely to be Asian, less likely to be White, more likely to be Hispanic, more likely to live in the West and Northeast, and less likely to live in the South. Compared to their native-born counterparts, immigrant adults were 3 years younger, and have lower rates of chronic disease.

### Logistic Regressions

Table 2 shows the results of two logistic regressions in the pooled sample with educational attainment as the predictor and psychological distress, SRH, and CDs as the outcomes (dependent variables). *Model 1* only included the main effects. However, *Model 2* added the interaction terms between immigration status with educational attainment.

**Table 2:** Logistic regressions in the pooled sample (33,672).

	**Psychological distress**				**Self-rated health**				**Chronic diseases**			
	**OR**	**95% CI**		***p***	**OR**	**95% CI**		***p***	**OR**	**95% CI**		***p***
**Main effect model**												
Immigrant	0.88	0.80	0.96	0.004	0.78	0.70	0.87	<0.001	0.58	0.54	0.63	<0.001
Hispanic	0.85	0.78	0.93	<0.001	1.18	1.06	1.32	0.002	0.92	0.85	1.00	0.043
Race				<0.001				<0.001				<0.001
Black only	0.96	0.88	1.04	0.312	1.65	1.51	1.80	<0.001	1.18	1.09	1.27	<0.001
Native American/Alaska Native only	1.40	1.12	1.76	0.004	1.66	1.28	2.14	<0.001	1.38	1.10	1.73	0.006
Asian only	0.77	0.67	0.89	<0.001	1.04	0.87	1.24	0.674	0.96	0.85	1.08	0.496
Multiple race	1.48	1.25	1.76	<0.001	1.79	1.47	2.19	<0.001	1.38	1.16	1.64	<0.001
Race group not releasable	0.96	0.58	1.60	0.890	0.35	0.13	0.97	0.044	0.81	0.52	1.28	0.367
Gender (male)	0.72	0.68	0.76	<0.001	0.99	0.93	1.06	0.757	0.97	0.92	1.02	0.274
Region												
West												
Northeast	1.06	0.97	1.15	0.197	0.97	0.88	1.08	0.592	1.04	0.96	1.12	0.371
Midwest	0.96	0.89	1.04	0.363	0.91	0.82	1.00	0.051	1.03	0.96	1.11	0.456
South	0.98	0.91	1.05	0.570	1.11	1.02	1.21	0.013	1.02	0.96	1.09	0.475
Age	0.99	0.99	0.99	<0.001	1.03	1.03	1.03	<0.001	1.06	1.06	1.06	<0.001
Education				<0.001				<0.001				<0.001
Less than 12 years												
12 years	0.71	0.65	0.77	<0.001	0.51	0.46	0.55	<0.001	0.85	0.78	0.93	<0.001
13–15 years	0.66	0.60	0.72	<0.001	0.43	0.39	0.47	<0.001	0.90	0.82	0.99	0.022
16+ years	0.44	0.40	0.48	<0.001	0.19	0.17	0.21	<0.001	0.70	0.64	0.77	<0.001
Constant	0.92			0.183	0.09			<0.001	0.12			<0.001
**Interaction model**												
Immigrant	0.79	0.68	0.92	0.002	0.67	0.58	0.79	<0.001	0.66	0.57	0.77	<0.001
Hispanic	0.87	0.79	0.95	0.002	1.21	1.09	1.35	0.001	1.01	0.92	1.10	0.859
Race				<0.001				<0.001				<0.001
Whites only												
Black only	0.95	0.88	1.03	0.250	1.64	1.50	1.79	<0.001	1.37	1.27	1.48	<0.001
Native American/Alaska Native only	1.39	1.11	1.75	0.005	1.65	1.28	2.14	<0.001	1.35	1.06	1.70	0.014
Asian only	0.74	0.64	0.86	<0.001	1.01	0.84	1.20	0.956	1.06	0.94	1.21	0.350
Multiple race	1.48	1.25	1.75	<0.001	1.79	1.46	2.19	<0.001	1.26	1.06	1.51	0.010
Race group not releasable	0.97	0.59	1.61	0.918	0.35	0.13	0.98	0.045	1.01	0.62	1.64	0.979
Gender (male)	0.72	0.68	0.76	<0.001	0.99	0.93	1.05	0.701	1.17	1.12	1.24	<0.001
Region												
West												
Northeast	1.06	0.97	1.15	0.201	0.97	0.88	1.07	0.548	1.05	0.97	1.14	0.219
Midwest	0.96	0.89	1.04	0.323	0.90	0.82	1.00	0.041	1.10	1.02	1.19	0.012
South	0.98	0.91	1.05	0.498	1.10	1.02	1.20	0.020	1.16	1.08	1.24	<0.001
Age	0.99	0.99	0.99	<0.001	1.03	1.03	1.03	<0.001	1.07	1.07	1.07	<0.001
Education				<0.001				<0.001				<0.001
Less than 12 years												
12 years	0.69	0.62	0.76	<0.001	0.48	0.44	0.54	<0.001	0.84	0.76	0.94	0.002
13–15 years	0.64	0.58	0.71	<0.001	0.41	0.37	0.46	<0.001	0.86	0.78	0.96	0.008
16+ years	0.41	0.37	0.46	<0.001	0.18	0.16	0.20	<0.001	0.68	0.61	0.75	<0.001
Education × immigrant				0.009				0.056				0.053
12 years × immigrant	1.04	0.85	1.28	0.699	1.17	0.94	1.46	0.154	1.09	0.89	1.33	0.402
13–15 Years × immigrant	1.03	0.83	1.28	0.782	1.24	0.97	1.58	0.086	1.06	0.86	1.31	0.558
16+ Years × immigrant	1.36	1.11	1.67	0.003	1.39	1.08	1.79	0.010	1.28	1.06	1.54	0.011
Constant	0.95			0.451	0.09			<0.001	0.04			<0.001

Based on *Model 1*, high educational attainment was inversely associated with psychological distress (OR = 0.71, 95% CI = 0.65–0.77; *p* ≤ 0.001 for 12 years, OR = 0.66, 95% CI = 0.60–0.72; *p* ≤ 0.001 for 13–15 years, OR = 0.44, 95% CI = 0.40–0.48; *p* ≤ 0.001 for 16 + years.), SRH (OR = 0.51, 95% CI = 0.46–0.55; *p* ≤ 0.001 for 12 years, OR = 0.43, 95% CI = 0.39–0.47; *p* ≤ 0.001 for 13–15 years, OR = 0.19, 95% CI = 0.17–0.21; *p* ≤ 0.001 for 16 + years), and CDs (OR = 0.85, 95% CI = 0.78–0.93; *p* ≤ 0.001 for 12 years, OR = 0.90, 95% CI = 0.82–0.99; *p* = 0.022 for 13–15 Years; OR = 0.70, 95% CI = 0.64–0.77; *p* ≤ 0.001 for 16 + years). *Model 2*, however, revealed a statistically significant interaction between educational attainment and immigration on psychological distress (OR = 1.36; 95% CI = 1.11–1.67; *p* = 0.003 for 16 + Years × immigrant term), SRH (OR = 1.36; 95% CI = 1.11–1.67; *p* = 0.003 for 16 + Years × immigrant term), and CDs (OR = 1.36; 95% CI = 1.11– 1.67; *p* = 0.003 for 16 + Years × immigrant term). The model suggested that the protective effects of educational attainment against psychological distress, SRH, and CDs are all smaller for immigrant than native-born adults (Table 2).

### Logistic Regressions

Table 3 shows the results of two logistic regressions in native-born (*Model 3*) and one in immigrant (*Model 4*) adults for each outcome. In these models, educational attainment was the predictor and either psychological distress, SRH, and CDs were the outcome (dependent variable). Based on *Model 3*, high educational attainment was inversely associated with psychological distress, SRH, and CDs for native-born adults. *Model 3* also showed some protective effects of educational attainment on psychological distress, SRH, and CDs for immigrant adults. These protective effects were all larger for native-born than immigrant adults (Table 3).

**Table 3:** Logistic regressions in native-born and immigrant adults.

	**Psychological distress**				**Self-rated health**				**Chronic diseases**			
	**OR**	**95% CI**		***p***	**OR**	**95% CI**		***p***	**OR**	**95% CI**		***p***
**Native born**												
Hispanic	0.85	0.76	0.94	0.002	1.08	0.95	1.23	0.257	1.00	0.90	1.11	0.991
Race				<0.001				<0.001				<0.001
White only												
Black only	0.97	0.89	1.06	0.476	1.66	1.52	1.82	<0.001	1.40	1.29	1.52	<0.001
Native American/Alaska	1.36	1.06	1.76	0.017	1.47	1.09	1.97	0.012	1.44	1.10	1.87	0.008
Native only												
Asian only	0.77	0.61	0.98	0.036	1.05	0.78	1.43	0.732	1.12	0.90	1.39	0.313
Multiple race	1.50	1.25	1.80	<0.001	1.89	1.53	2.34	<0.001	1.35	1.11	1.63	0.002
Race group not releasable	0.46	0.19	1.10	0.080	0.00	0.00		0.997	1.15	0.59	2.25	0.680
Gender (male)	0.72	0.68	0.77	<0.001	1.03	0.96	1.11	0.337	1.19	1.12	1.26	<0.001
Region	1.00	0.91	1.10	0.940	0.88	0.78	0.99	0.034	1.06	0.97	1.16	0.221
West												
Northeast	0.94	0.86	1.02	0.156	0.94	0.85	1.05	0.266	1.16	1.07	1.26	<0.001
Midwest	0.97	0.89	1.05	0.384	1.17	1.06	1.28	0.001	1.24	1.15	1.34	<0.001
South	0.99	0.99	0.99	<0.001	1.02	1.02	1.03	<0.001	1.07	1.07	1.07	<0.001
Education				<0.001				<0.001				<0.001
Less than 12 years												
12 years	0.69	0.62	0.76	<0.001	0.48	0.44	0.53	<0.001	0.85	0.76	0.95	0.003
13–15 years	0.63	0.57	0.70	<0.001	0.40	0.36	0.45	<0.001	0.87	0.78	0.97	0.009
16+ years	0.41	0.37	0.45	<0.001	0.17	0.16	0.20	<0.001	0.68	0.62	0.76	<0.001
Constant	1.08			0.279	0.11			<0.001	0.04			<0.001
**Immigrant**												
Hispanic	0.90	0.75	1.08	0.257	1.65	1.31	2.07	<0.001	0.97	0.82	1.16	0.753
Race				0.001				0.057				0.867
White only												
Black only	0.80	0.62	1.02	0.075	1.11	0.82	1.50	0.514	1.06	0.85	1.32	0.604
Native American/Alaska	1.44	0.87	2.38	0.160	2.42	1.41	4.14	0.001	1.03	0.61	1.74	0.913
Native only												
Asian only	0.72	0.58	0.88	0.002	1.00	0.76	1.30	0.991	0.93	0.77	1.12	0.438
Multiple race	1.25	0.76	2.04	0.382	1.07	0.56	2.04	0.836	0.85	0.51	1.40	0.521
Race group not releasable	1.96	1.02	3.78	0.044	1.00	0.34	2.88	0.996	0.88	0.43	1.80	0.723
Gender (male)	0.70	0.62	0.80	<0.001	0.79	0.68	0.93	0.005	1.14	1.01	1.29	0.031
Region	1.28	1.07	1.54	0.007	1.30	1.05	1.60	0.015	1.06	0.89	1.26	0.504
West												
Northeast	1.11	0.89	1.39	0.344	0.68	0.50	0.94	0.018	0.87	0.70	1.07	0.173
Midwest	1.00	0.85	1.17	0.966	0.80	0.66	0.97	0.025	0.89	0.77	1.03	0.108
South	1.00	1.00	1.01	0.427	1.05	1.05	1.06	<0.001	1.08	1.07	1.08	<0.001
Education				<0.001				<0.001				0.851
Less than 12 years												
12 years	0.75	0.62	0.89	0.002	0.63	0.52	0.78	<0.001	0.95	0.80	1.13	0.588
13–15 years	0.72	0.58	0.88	0.001	0.59	0.47	0.75	<0.001	0.96	0.79	1.16	0.668
16+ years	0.61	0.50	0.73	<0.001	0.31	0.24	0.40	<0.001	0.92	0.77	1.10	0.378
Constant	0.38			<0.001	0.02			<0.001	0.02			<0.001

## Discussion

The current study supports the finding that all educational credentials are associated with lower odds of psychological distress, poor SRH, and CDs; however, the protective effects of 16+ years of education are stronger for native-born than immigrant adults in the US.

The observation that as educational attainment credentials are gained, odds of psychological distress, poor SRH, and CDs decreases, but 16+ years of education better promote health for native-born than immigrant adults in the US, is an extension of previous literature on MDRs (28, 29). Previous research found that the association between educational attainment and a wide range of physical health outcomes such as self-rated health (32, 37, 107), CDs (35, 108, 109), depression (110, 111), suicide (31), obesity (33, 34), disability (112), and mortality (36) was weaker in Black and Hispanic relative to Non-Hispanic White people. It is also in line with the observations that education generates less health for LGBT than non-LGBT individuals (41–43).

The robust and consistent nature of the MDRs suggests that differences in education and health may be due to the function and structure of society. US social institutions differentially treat people based on their color, race, ethnicity, class, heritage, and nativity (113). These result in systemic marginalization of non-majority groups. Such marginalization reduces people's chance of full participation and full benefit from resources that are available to them. Racism, xenophobia, and nationalism are embedded in the social fabric of the US society and reduce immigrant adults' ability to fully benefit their own human capital and turn their resources into tangible outcomes. As a result, they show less than expected benefits in the presence of educational attainment (28, 29).

It seems that it is not just educational attainment (32) but also income (109), occupation (36), and marital status (114) that tend to generate better health for the majority than marginalized people. These MDRs are not just for psychological distress (107), SRH (12, 32, 107), and CDs (35, 108, 109) but for obesity (33, 34), vaping (115), smoking (40, 42, 116–118), drinking (44, 119), diet (120), exercise (11), hospitalization (121), and mortality (36). Thus, MDRs are neither specific to SES indicators nor to any health outcomes. They are seen for mental health, behaviors, and physical health. These universal patterns of MDRs suggest that they are due to upstream social processes rather than group behaviors.

The observed MDRs of educational attainment on the protective effects of high educational attainment on psychological distress, SRH, and CDs may be because, similar to poverty, race, and ethnicity, immigration status shapes life chances and health. Immigrant adults generally have better health than US-born individuals, despite having lower education. We would assume that additional education would significantly augment immigrant health. However, the diminished health return of education suggest that additional education may not protect immigrant individuals from exposure to discrimination, risky work conditions, and having limited access to quality healthcare (122). We found that relative to highly educated native Americans, highly educated immigrant adults remain at an increased risk of health problems such as psychological distress, poor SRH, and CDs. Clinicians and healthcare providers should be cognizant that immigrant adults, regardless of education, are exposed to structural barriers such as immigration laws, labor market laws, and residential segregation that potentially reduce the health gain of social upward mobility.

### Area of Future Research

First of all, the results reported here are exclusively relevant to the US context. Thus, there is a need to conduct similar studies in other countries. Immigration is not a simple and singular variable such as age, gender, or even nationality. Immigration is a proxy of culture, lived experiences, life history, and marginalization. That said, research has continuously shown that one size does not fit all regarding immigrant adults' health status. By that we mean that these factors contribute to health profile of immigrant adults across education levels. In this study immigration was conceptualized as an over-simplistic dichotomous variable due to the data that were available to us. This should be addressed in future research; however, here we partly discuss the intersection of SES, immigration, and health.

While this study relied on MDRs (10, 28, 29), which predominantly focused on diminished returns of SES indicators for marginalized people (100), other frameworks and theories could also help us understand the involved processes that may explain our findings. One related framework is intersectionality (19, 20, 123). Supporting the intersectionality framework (19, 20, 123), we found different predictors of health of population sub-groups based on the intersection of immigration status and SES. That is, in the US, intersections of immigration status and SES, rather than each of them separately, have implications for the health of population. In line with the intersectionality theory, we found multiplicative rather than additive effects of immigration status and SES on health. Each intersectional group has a unique set of social identities, resources, risk factors, and social identities. As such, they differ in how their visible and invisible identity markers clash with systems of privilege and marginalization in the US. Acculturation is also another framework that can advance the existing knowledge on the observed MDRs and make sense of the results. Immigrant people integrate to the host country and become more similar to them as time passes since immigration (68, 70, 124). We, however, did not have data on length of time post immigration (67, 68, 125, 126). As individuals lose their attachment to their culture and original identity, and as they acquire the behavioral characters of the host country, their health status becomes more similar to the native-born people (67, 68, 125, 126).

As a result of Xenophobia, and as seen as “foreigners,” immigrant people may experience high levels of hate crime, discrimination, and prejudice (95), which result in marginalization and social isolation. Discrimination is one of the major factors in explaining poor health of immigrant people (16, 45, 47, 49, 127). Immigrant populations face additional difficulties to enter the labor market and secure high-quality jobs, as they compete with native-born people (128). Many immigrant adults work in low-paying jobs and provide uncompensated labor (129). Finally, some immigrant people live in ethnic enclaves and highly segregated areas, and some send money back to their country (130).

The observed MDRs (interaction between education and immigration status) can be attributed to discrimination in the education system (131). Immigrant and native-born individuals do not have the same opportunity for education in the US. They are also not equally treated by the education system (132). Discrimination, second tier education, and systematic marginalization might uniquely contribute to the observed MDRs in the immigration. The labor market may also undervalue their education (133), if education is achieved in their own country not the US (134).

We argue that immigrant individuals are also racialized in the US; however, the effect of immigration status depends on SES, as shown by this study. While the focus of this study was on how immigration status and SES intersect, most previous literature on the intersectionality has focused on race, ethnicity, gender, sexual orientation, and socioeconomic status (18–20). As such, this study extends the previous work to one of the less frequently studied aspects of intersectionality of social identities. Still, the intersectionality framework helps us understand the results as it suggests that the complex and overlapping layers of inequities expose subgroups of the population to unique health risks. In this view, life experiences, sets of exposures, and vulnerabilities are not a function of each by combination of various social identities, as documented by the interactions between various social identities (multiplicative rather than additive effects). Thus, there is always a need to understand the differences in the health status of groups based on the intersections of nativity and SES, as shown here. The same applied to the intersections of race, ethnicity, gender, age, physical ability, and sexual orientation.

### Implications

The result of this study has some policy implications. First, the solution to eliminate health inequalities should not be limited to closing the SES gap across social groups. While SES and SDOH are important, it is not just their access but utility and the degree by which they become outcomes. To eliminate health inequality between immigrant and native-born people, it is essential to equalize the health return of educational attainment. Thus, we need to go beyond equal access to education but equality in the returns of educational attainment for social groups. Specific policies and programs should help immigrant adults to more effectively mobilize and leverage their available human capital to gain tangible outcomes. When a policy is designed and implemented, these MDRs should be investigated. These types of evidence suggest that MDRs should be targeted as a part of equitable goals.

### Limitations

The current results should be interpreted with the methodological limitations in mind. First, any cross-sectional study is limited in drawing causal inferences. We cannot rule out that excessive health problems would influence social mobility and the ability to attain education. Thus, reverse causality cannot be ruled out in this study. Prospective research is needed to better understand the association between immigration status, educational attainment, and health. Furthermore, future research should examine the mechanisms by which MDRs of educational attainment emerge. We did not have access to the country of origin, or whether education was attained in the US or the origin country. There is a need to compare immigrant subgroups from Asia, Africa, and Latino countries as each culture may differently adopt US culture. We also did not control for the type of college education of other covariates such as wealth or being a first-generation college student. Future research should replicate and validate these findings using longitudinal data, with a more comprehensive list of measures such as country of origin and details of educational attainment. Future research may also include contextual factors such as ethnic composition, SES, or density of resources at the neighborhood level. It is likely that highly educated immigrant individuals still live in areas with low availability of green spaces and parks that are essential for health.

### Application of Single-Item Measures

Although this study has some strengths, such as a large n and representative sample, it was limited in terms of measurement. While single item is commonly used for overall and physical health, single-item measure of self-rated psychological health is limited in their ability to assess mental health. Thus, we compensated this weakness by including three outcomes that could cover various domains of both physical and mental health. Single-item measures are widely used for overall, general, and physical health (135–143). They are used, but less common to evaluate self-rated psychological health (144–150). Still, these measures reflect mental health, depression, and anxiety, and psychological distress. As they are cost efficient, easy to administer, reliable, valid, and easily understood by the people, they are commonly used in large-scale national surveys. They may, however, be prone to differential validity across demographic groups (151–154). That means, these single items may mean different things for different groups (151–154). It is, however, very unlikely that bias shapes a spurious association regardless of the domain of health. Similarity of the results across outcomes provided additional support and confidence for our findings. Thus, despite our measurement limitations, the observed MDRs seem to be robust. Still, at interpretation of the results, readers should be aware of the measurement problem in this paper.

Future research should find large and balanced sample sizes of ethnic groups and test for replication of these findings across ethnic groups. The reason we did not run ethnic-specific models was statistical power (e.g., few immigrant Black people) and unequal variance of SES across ethnic groups. Combining all or several years of NHIS data or General Health Survey (GSS) would probably have enough statistical power for such future investigations.

## Conclusion

In the US, educational attainment better reduces the odds of psychological distress, poor SRH, CDs, and obesity for native-born than immigrant adults. Thus, at least some of the health disparities in the US between immigrant and native-born people are due to inequalities in marginal returns of educational attainment for immigrant population.

## Data Availability Statement

The datasets generated for this study are available here: https://www.cdc.gov/nchs/nhis/nhis_2015_data_release.htm.

## Ethics Statement

All participants signed written consent. The NHIS protocol was approved by the CDC Institutional Review Board (IRB).

## Author Contributions

SA was responsible for the conceptualization, data analysis, preparation of the first draft, and revision. SC and MB were in charge of the revision of the manuscript and approved the final draft. All authors contributed to the article and approved the submitted version.

## Conflict of Interest

The authors declare that the research was conducted in the absence of any commercial or financial relationships that could be construed as a potential conflict of interest.

## References

[1] Marmot M. Social determinants of health inequalities. Lancet. (2005) 365:1099–104. 10.1016/S0140-6736(05)71146-6 15781105

[2] Marmot M The Status Syndrome: How Social Standing Affects Our Health and Longevity. London: Bloomsbury Press (2004).

[3] Marmot M. Economic and social determinants of disease. Bull World Health Organ. (2001) 79:988–9. Available online at: https://www.ncbi.nlm.nih.gov/pmc/articles/PMC2566682/11693982 PMC2566682

[4] Hudson DL Puterman E Bibbins-Domingo K Matthews KA Adler NE. Race, life course socioeconomic position, racial discrimination, depressive symptoms and self-rated health. Soc Sci Med. (2013) 97:7–14. 10.1016/j.socscimed.2013.07.031 24161083

[5] Murcia M Chastang JF Niedhammer I. Educational inequalities in major depressive and generalized anxiety disorders: results from the French national SIP study. Soc Psychiatry Psychiatr Epidemiol. (2015) 50:919–28. 10.1007/s00127-015-1010-9 25605025

[6] Phillips JA Hempstead K. Differences in U.S. Suicide Rates by Educational Attainment, 2000-2014. Am J Prev Med. (2017) 53:e123–30. 10.1016/j.amepre.2017.04.010 28756896

[7] Merz EC Tottenham N Noble KG. Socioeconomic status, amygdala volume, and internalizing symptoms in children and adolescents. J Clin Child Adolesc Psychol. (2018) 47:312–23. 10.1080/15374416.2017.1326122 28574722 PMC6116521

[8] Ursache A Merz EC Melvin S Meyer J Noble KG. Socioeconomic status, hair cortisol and internalizing symptoms in parents and children. Psychoneuroendocrinology. (2017) 78:142–50. 10.1016/j.psyneuen.2017.01.020 28199857 PMC5421817

[9] Assari S. Race, education attainment, and happiness in the United States. Int. J. Epidemiol. Res. (2019) 6:76. 10.15171/ijer.2019.14 31363495 PMC6666429

[10] Assari S. Socioeconomic determinants of systolic blood pressure; minorities' diminished returns. J. Health Econ Dev. (2019) 1:1–11. 31428747

[11] Assari S. Educational attainment and exercise frequency in american women; blacks' diminished returns. Women's Health Bulletin. (2019) 6:e87413. 10.5812/whb.87413 31552286 PMC6757331

[12] Assari S. Ethnicity, educational attainment, and physical health of older adults in the United States. Aging Med. (2019) 2:104–11. 10.1002/agm2.12050 31608316 PMC6788632

[13] Assari S. Parental educational attainment and academic performance of american college students; blacks' diminished returns. J Health Econ Dev. (2019) 1:21–31. 31372601 PMC6673665

[14] Abe-Kim J Takeuchi DT Hong S Zane N Sue S Spencer MS. Use of mental health-related services among immigrant and US-born Asian Americans: results from the National Latino and Asian American Study. Am J Public Health. (2007) 97:91–8. 10.2105/AJPH.2006.098541 17138905 PMC1716256

[15] Alegria M Canino G Shrout PE Woo M Duan N Vila D. Prevalence of mental illness in immigrant and non-immigrant U.S. Latino groups. Am J Psychiatry. (2008) 165:359–69. 10.1176/appi.ajp.2007.07040704 18245178 PMC2712949

[16] Fortuna LR Alvarez K Ramos Ortiz Z Wang Y Alegria XM Cook BL. Mental health, migration stressors and suicidal ideation among latino immigrants in Spain and the United States. Eur Psychiatry. (2016) 36:15–22. 10.1016/j.eurpsy.2016.03.001 27311103 PMC5500916

[17] Fortuna LR Porche MV Alegria M Political violence, psychosocial trauma, and the context of mental health services use among immigrant Latinos in the United States. Ethn Health. (2008) 13:435–63. 10.1080/13557850701837286 18850369 PMC2771411

[18] Bowleg L When Black+ lesbian+ woman≠ Black lesbian woman: the methodological challenges of qualitative and quantitative intersectionality research. Sex Roles. (2008) 59:312–25. 10.1007/s11199-008-9400-z

[19] Cole ER Intersectionality and research in psychology. Am Psychol. (2009) 64:170 10.1037/a0014564 19348518

[20] Carbado DW Crenshaw KW Mays VM Tomlinson B. Intersectionality: mapping the movements of a theory. Du Bois Rev. (2013) 10:303–12. 10.1017/S1742058X13000349 25285150 PMC4181947

[21] Assari S. Cross-country differences in the additive effects of socioeconomics, health behaviors and medical comorbidities on disability among older adults with heart disease. J Tehran Heart Cent. (2015) 10:24–33. 10.1186/2251-6581-13-75 26157460 PMC4494516

[22] Assari S Lankarani MM. Does multi-morbidity mediate the effect of socioeconomics on self-rated health? Cross-country differences. Int J Prev Med. (2015) 6:85. 10.4103/2008-7802.164413 26445632 PMC4587073

[23] Assari S. Cross-country variation in additive effects of socio-economics, health behaviors, and comorbidities on subjective health of patients with diabetes. J Diabetes Metab Disord. (2014) 13:36. 10.1186/2251-6581-13-36 24559091 PMC3984018

[24] Assari S Lankarani RM Lankarani MM Cross-country differences in the association between diabetes and disability. J Diabetes Metab Disord. (2014) 13:3 10.1186/2251-6581-13-3 24393171 PMC3927767

[25] Hudson DL Bullard KM Neighbors HW Geronimus AT Yang J Jackson JS. Are benefits conferred with greater socioeconomic position undermined by racial discrimination among African American men? J Mens Health. (2012) 9:127–36. 10.1016/j.jomh.2012.03.006 22707995 PMC3371660

[26] Hudson DL Neighbors HW Geronimus AT Jackson JS. The relationship between socioeconomic position and depression among a US nationally representative sample of African Americans. Soc Psychiatry Psychiatr Epidemiol. (2012) 47:373–81. 10.1007/s00127-011-0348-x 21293845 PMC3279642

[27] Hudson DL Neighbors HW Geronimus AT Jackson JS. Racial discrimination, john henryism, and depression among African Americans. J Black Psychol. (2016) 42:221–43. 10.1177/0095798414567757 27529626 PMC4903152

[28] Assari S Health disparities due to diminished return among black Americans: public policy solutions. Social Issues and Policy Review. (2018) 12:112–45. 10.1111/sipr.12042

[29] Assari S. Unequal gain of equal resources across racial groups. Int J Health Policy Manag. (2017) 7:1–9. 10.15171/ijhpm.2017.90 29325397 PMC5745862

[30] Assari S Mistry R Educational attainment and smoking status in a National Sample of American adults; evidence for the blacks' diminished return. Int J Environ Res Public Health. (2018) 15:763 10.3390/ijerph15040763 PMC5923805 29659482

[31] Assari S Schatten HT Arias SA Miller IW Camargo CA Boudreaux ED Higher educational attainment is associated with lower risk of a future suicide attempt among non-hispanic whites but not non-hispanic blacks. J Racial Ethn Health Disparities. (2019) 6:1001–10. 10.1007/s40615-019-00601-z 31278625 PMC6739140

[32] Assari S. Blacks' diminished return of education attainment on subjective health; mediating effect of income. Brain Sci. (2018) 8:176. 10.3390/brainsci8090176 30213135 PMC6162786

[33] Assari S Family income reduces risk of obesity for white but not black children. Children. (2018) 5:73 10.3390/children5060073 PMC6025246 29890778

[34] Assari S Thomas A Caldwell CH Mincy RB. Blacks' diminished health return of family structure and socioeconomic status; 15 years of follow-up of a national urban sample of youth. J Urban Health. (2018) 95:21–35. 10.1007/s11524-017-0217-3 29230628 PMC5862702

[35] Assari S Moghani Lankarani M. Poverty status and childhood asthma in white and black families: national survey of children's health. Healthcare. (2018) 6:62. 10.3390/healthcare6020062 29895767 PMC6023379

[36] Assari S Lankarani MM Race and urbanity alter the protective effect of education but not income on mortality. Front Public Health. (2016) 4:100 10.3389/fpubh.2016.00100 27242992 PMC4873510

[37] Assari S. Socioeconomic status and self-rated oral health; diminished return among hispanic whites. Dent J. (2018) 6:11. 10.3390/dj6020011 29695074 PMC6023433

[38] Assari S Bazargan M. Educational attainment and self-rated oral health among american older adults: hispanics' diminished returns. Dentistry J. (2019) 7:97. 10.3390/dj7040097 31581515 PMC6960509

[39] Assari S Boyce S Bazargan M Caldwell CH. Mathematical performance of American Youth: diminished returns of educational attainment of Asian-American parents. Educ Sci. (2020) 10:32. 10.3390/educsci10020032 32201681 PMC7083587

[40] Assari S Bazargan M. Protective effects of educational attainment against cigarette smoking; diminished returns of American Indians and Alaska Natives in the National Health Interview Survey. Int J Travel Med Global Health. (2019) 7:105–10. 10.15171/ijtmgh.2019.22 31772950 PMC6879009

[41] Assari S Bazargan M. Educational attainment and subjective health and well-being; diminished returns of lesbian, gay, and bisexual individuals. Behav Sci. (2019) 9:90. 10.3390/bs9090090 31443340 PMC6770696

[42] Assari S Bazargan M. Education level and cigarette smoking: diminished returns of lesbian, gay and bisexual individuals. Behav Sci. (2019) 9:103. 10.3390/bs9100103 31554198 PMC6826997

[43] Assari S. Education attainment and obesitydifferential returns based on sexual orientation. Behav Sci. (2019) 9:16. 10.3390/bs9020016 30699932 PMC6406256

[44] Assari S Farokhnia M Mistry R. Education attainment and alcohol binge drinking: diminished returns of hispanics in Los Angeles. Behav Sci. (2019) 9:9. 10.3390/bs9010009 30646592 PMC6359422

[45] Cobb CL Meca A Branscombe NR Schwartz SJ Xie D Zea MC. Perceived discrimination and well-being among unauthorized hispanic immigrants: the moderating role of ethnic/racial group identity centrality. Cultur Divers Ethnic Minor Psychol. (2018) 25:280–7. 10.1037/cdp0000227 30284850

[46] Fischer S Nater UM Strahler J Skoluda N Dieterich L Oezcan O. Psychobiological impact of ethnic discrimination in Turkish immigrants living in Germany. Stress. (2017) 20:167–74. 10.1080/10253890.2017.1296430 28276806

[47] Flippen CA Parrado EA. Perceived discrimination among Latino immigrants in new destinations: the case of Durham, North Carolina. Sociol Perspect. (2015) 58:666–85. 10.1177/0731121415574397 26848208 PMC4734395

[48] Straiton ML Aambo AK Johansen R. Perceived discrimination, health and mental health among immigrants in Norway: the role of moderating factors. BMC Public Health. (2019) 19:325. 10.1186/s12889-019-6649-9 30894173 PMC6425660

[49] Yoo HC Gee GC Takeuchi D. Discrimination and health among Asian American immigrants: disentangling racial from language discrimination. Soc Sci Med. (2009) 68:726–32. 10.1016/j.socscimed.2008.11.013 19095340 PMC3897711

[50] Bostean G. Does selective migration explain the Hispanic paradox? A comparative analysis of Mexicans in the U.S. and Mexico. J Immigr Minor Health. (2013) 15:624–35. 10.1007/s10903-012-9646-y 22618355 PMC3901783

[51] McDonald JT Kennedy S. Insights into the 'healthy immigrant effect': health status and health service use of immigrants to Canada. Soc Sci Med. (2004) 59:1613–27. 10.1016/j.socscimed.2004.02.004 15279920

[52] Franzini L Ribble JC Keddie AM. Understanding the Hispanic paradox. Ethn Dis. (2001) 11:496–518. 11572416

[53] Markides KS Eschbach K. Aging, migration, and mortality: current status of research on the Hispanic paradox. J Gerontol B Psychol Sci Soc Sci. (2005) 60:68–75. 10.1093/geronb/60.Special_Issue_2.S68 16251594

[54] McCarthy M. CDC report confirms “Hispanic paradox”. BMJ. (2015) 350:h2467. 10.1136/bmj.h2467 25952797

[55] Balcazar AJ Grineski SE Collins TW. The Hispanic health paradox across generations: the relationship of child generational status and citizenship with health outcomes. Public Health. (2015) 129:691–7. 10.1016/j.puhe.2015.04.007 26002345 PMC4475672

[56] Shor E Roelfs D Vang ZM. The “Hispanic mortality paradox” revisited: META-analysis and meta-regression of life-course differentials in Latin American and Caribbean immigrants' mortality. Soc Sci Med. (2017) 186:20–33. 10.1016/j.socscimed.2017.05.049 28577458

[57] Calvo R Carr DC Matz-Costa C. Another paradox? The life satisfaction of older hispanic immigrants in the United States. J Aging Health. (2017) 29:3–24. 10.1177/0898264315624901 26772911

[58] Schoenthaler AM. Cardiovascular health in Hispanics/Latinos: a reexamination of the Hispanic paradox. J Clin Hypertens. (2017) 19:114–5. 10.1111/jch.12941 27860136 PMC7576677

[59] Valles SA. The challenges of choosing and explaining a phenomenon in epidemiological research on the “Hispanic Paradox”. Theor Med Bioeth. (2016) 37:129–48. 10.1007/s11017-015-9349-1 26754488

[60] Lerman-Garber I Villa AR Caballero E. Diabetes and cardiovascular disease. Is there a true Hispanic paradox? Rev Invest Clin. (2004) 56:282–96. 15612509

[61] Molina E Haas R del Rincon I Battafarano DF Restrepo JF Escalante A. Does the “Hispanic paradox” occur in rheumatoid arthritis? Survival data from a multiethnic cohort. Arthritis Care Res. (2014) 66:972–9. 10.1002/acr.22254 24339449 PMC4051868

[62] Thomson EF Nuru-Jeter A Richardson D Raza F Minkler M. The Hispanic Paradox and older adults' disabilities: is there a healthy migrant effect? Int J Environ Res Public Health. (2013) 10:1786–814. 10.3390/ijerph10051786 23644828 PMC3709349

[63] Crimmins EM Kim JK Alley DE Karlamangla A Seeman T. Hispanic paradox in biological risk profiles. Am J Public Health. (2007) 97:1305–10. 10.2105/AJPH.2006.091892 17538054 PMC1913070

[64] Luke B Brown MB Misiunas RB Gonzalez-Quintero VH Nugent C van de Ven C. The hispanic paradox in twin pregnancies. Twin Res Hum Genet. (2005) 8:532–7. 10.1375/twin.8.5.532 16212843

[65] Drummond MB. The hispanic paradox unraveled? Am J Respir Crit Care Med. (2011) 184:1222–3. 10.1164/rccm.201109-1684ED 22162880 PMC3262040

[66] Albrecht SS Gordon-Larsen P. Ethnic differences in body mass index trajectories from adolescence to adulthood: a focus on Hispanic and Asian subgroups in the United States. PLoS ONE. (2013) 8:e72983. 10.1371/journal.pone.0072983 24039835 PMC3764158

[67] Haderxhanaj LT Dittus PJ Loosier PS Rhodes SD Bloom FR Leichliter JS. Acculturation, sexual behaviors, and health care access among Hispanic and non-Hispanic white adolescents and young adults in the United States, 2006-2010. J Adolesc Health. (2014) 55:716–9. 10.1016/j.jadohealth.2014.06.018 25156896 PMC5774013

[68] Nwadiora E McAdoo H. Acculturative stress among Amerasian refugees: gender and racial differences. Adolescence. (1996) 31:477–87. 8726905

[69] Bos V Kunst AE Garssen J Mackenbach JP. Duration of residence was not consistently related to immigrant mortality. J Clin Epidemiol. (2007) 60:585–92. 10.1016/j.jclinepi.2006.08.010 17493514

[70] Chang E Chan KS Han HR. Effect of acculturation on variations in having a usual source of care among Asian Americans and non-Hispanic whites in California. Am J Public Health. (2015) 105:398–407. 10.2105/AJPH.2014.301950 25033147 PMC4318297

[71] Killoren SE Zeiders KH Updegraff KA Umana-Taylor AJ. The sociocultural context of mexican-origin pregnant adolescents' attitudes toward teen pregnancy and links to future outcomes. J Youth Adolesc. (2016) 45:887–99. 10.1007/s10964-015-0387-9 26573862 PMC4826852

[72] Assari S Lankarani MM. Discrimination and psychological distress: gender differences among Arab Americans. Front Psychiatry. (2017) 8:23. 10.3389/fpsyt.2017.00023 28265246 PMC5316930

[73] Wang Q. Health of the elderly migration population in China: benefit from individual and local socioeconomic status? Int J Environ Res Public Health. (2017) 14:370. 10.3390/ijerph14040370 28368314 PMC5409571

[74] Malmusi D Palencia L Ikram UZ Kunst AE Borrell C. Inequalities by immigrant status in depressive symptoms in Europe: the role of integration policy regimes. Soc Psychiatry Psychiatr Epidemiol. (2017) 52:391–8. 10.1007/s00127-017-1348-2 28194503

[75] Osypuk TL Diez Roux AV Hadley C Kandula NR. Are immigrant enclaves healthy places to live? The Multi-ethnic Study of Atherosclerosis. Soc Sci Med. (2009) 69:110–20. 10.1016/j.socscimed.2009.04.010 19427731 PMC2750873

[76] Xie Y Gough M. Ethnic enclaves and the earnings of immigrants. Demography. (2011) 48:1293–315. 10.1007/s13524-011-0058-8 21863367 PMC3226926

[77] Campbell JR Ethnic enclaves—Cul-de-sacs or conduits: differential aspirations in Greek American, Caucasian American, Latino, and Asian American neighborhoods in New York City. Int J Educ Res. (1994) 21:723–38. 10.1016/0883-0355(94)90045-0

[78] Weden MM Miles JNV Friedman E Escarce JJ Peterson C Langa KM. The hispanic paradox: race/ethnicity and nativity, immigrant enclave residence and cognitive impairment among older US Adults. J Am Geriatr Soc. (2017) 65:1085–91. 10.1111/jgs.14806 28369694 PMC5435528

[79] Von Behren J Abrahao R Goldberg D Gomez SL Setiawan VW Cheng I. The influence of neighborhood socioeconomic status and ethnic enclave on endometrial cancer mortality among Hispanics and Asian Americans/Pacific Islanders in California. Cancer Causes Control. (2018) 29:875–81. 10.1007/s10552-018-1063-7 30056614 PMC7466936

[80] Yang TC Shoff C Noah AJ Black N Sparks CS. Racial segregation and maternal smoking during pregnancy: a multilevel analysis using the racial segregation interaction index. Soc Sci Med. (2014) 107:26–36. 10.1016/j.socscimed.2014.01.030 24602968 PMC4029363

[81] Viruell-Fuentes EA Morenoff JD Williams DR House JS. Contextualizing nativity status, Latino social ties, and ethnic enclaves: an examination of the 'immigrant social ties hypothesis'. Ethn Health. (2013) 18:586–609. 10.1080/13557858.2013.814763 23947776 PMC4176765

[82] Barajas-Gonzalez RG Brooks-Gunn J. Income, neighborhood stressors, and harsh parenting: test of moderation by ethnicity, age, and gender. J Fam Psychol. (2014) 28:855–66. 10.1037/a0038242 25383794

[83] Altarriba J Bauer LM Counseling the hispanic client: cuban americans, mexican americans, and puerto ricans. J Counsel Dev. (1998) 76:389–96. 10.1002/j.1556-6676.1998.tb02697.x

[84] Shrout PE Canino GJ Bird HR Rubio-Stipec M Bravo M Burnam MA. Mental health status among Puerto Ricans, Mexican Americans, and non-Hispanic whites. Am J Community Psychol. (1992) 20:729–52. 10.1007/BF01312605 1302447

[85] Randolph WM Stroup-Benham C Black SA Markides KS. Alcohol use among Cuban-Americans, Mexican-Americans, and Puerto Ricans. Alcohol Health Res World. (1998) 22:265. 15706753 PMC6761886

[86] Keigher SM. America's most cruel xenophobia. Health Social Work. (1997) 22:232. 10.1093/hsw/22.3.232 9260087

[87] Yakushko O Xenophobia: understanding the roots and consequences of negative attitudes toward immigrants. Counseling Psychol. (2009) 37:36–66. 10.1177/0011000008316034

[88] Hjerm M National identities, national pride and xenophobia: a comparison of four Western countries. Acta Sociol. (1998) 41:335–47. 10.1080/00016999850080005

[89] Ghodsee K Left wing, right wing, everything: xenophobia, neo-totalitarianism, and populist politics in Bulgaria. Problems Post-Communism. (2008) 55:26–39. 10.2753/PPC1075-8216550303

[90] Inglehart R Norris P Trump and the xenophobic populist parties: the silent revolution in reverse. Perspectives Politics. (2017) 15:443–54. 10.1017/S1537592717000111

[91] Anderson-Nathe B Gharabaghi K (editors). Trending Rightward: Nationalism, Xenophobia, and the 2016 Politics of Fear. (2017). 10.1080/0145935X.2017.1277125

[92] Whitehead AL Perry SL Baker JO Make America Christian again: Christian nationalism and voting for Donald Trump in the 2016 presidential election. Sociol Religion. (2018) 79:147–71. 10.1093/socrel/srx070

[93] Macgregor-Bowles I Bowles DC. Trump, Brexit, Right-wing anti-globalisation, and an uncertain future for public health. AIMS Public Health. (2017) 4:139. 10.3934/publichealth.2017.2.139 29546210 PMC5689801

[94] Miller C Arcostanzo F Smith J Krasodomski-Jones A Wiedlitzka S Jamali R From brussels to Brexit: islamophobia, xenophobia, racism and reports of hateful incidents on twitter. Demos. (2016).

[95] Shively M Subramanian R Drucker O Edgerton J McDevitt J Farrell A Understanding trends in hate crimes against immigrants and Hispanic- Americans. (2014). Available online at: https://www.ncjrs.gov/pdffiles1/nij/grants/244755.pdf; http://hdl.handle.net/20.500.11990/1662 (accessed October 10, 2020).

[96] Bilge S Denis A Introduction: women, intersectionality and diasporas. J Intercultural Stud. (2010) 31:1–8. 10.1080/07256860903487653

[97] Haritatos J Mahalingam R James SA. John Henryism, self-reported physical health indicators, and the mediating role of perceived stress among high socio-economic status Asian immigrants. Soc Sci Med. (2007) 64:1192–203. 10.1016/j.socscimed.2006.10.037 17174456

[98] Mistry RS Biesanz JC Chien N Howes C Benner AD Socioeconomic status, parental investments, and the cognitive and behavioral outcomes of low-income children from immigrant and native households. Early Childhood Res Quarterly. (2008) 23:193–212. 10.1016/j.ecresq.2008.01.002

[99] Zsembik BA Fennell D. Ethnic variation in health and the determinants of health among Latinos. Soc Sci Med. (2005) 61:53–63. 10.1016/j.socscimed.2004.11.040 15847961

[100] Assari S. Income and mental well-being of middle-aged and Older Americans: immigrants' diminished returns. Int J Travel Med Global Health. (2020) 8:37–43. 10.34172/ijtmgh.2020.06 32266301

[101] Ho A Shih M Simon P. Hispanic paradox. Am J Public Health. (2007) 97:392–3. 10.2105/AJPH.2006.105320 17267705 PMC1805020

[102] Brown HL Chireau MV Jallah Y Howard D. The “Hispanic paradox”: an investigation of racial disparity in pregnancy outcomes at a tertiary care medical center. Am J Obstet Gynecol. (2007) 197:e197–9. 10.1016/j.ajog.2007.04.036 17689648

[103] Alegría M Sribney W Woo M Torres M Guarnaccia P. Looking beyond nativity: the relation of age of immigration, length of residence, and birth cohorts to the risk of onset of psychiatric disorders for Latinos. Res Hum Dev. (2007) 4:19–47. 10.1080/15427600701480980 19412354 PMC2675924

[104] Takeuchi DT Alegría M Jackson JS Williams DR. Immigration and mental health: Diverse findings in Asian, Black, and Latino populations. In: American Public Health Association. (2007). 97:11–2. 10.2105/AJPH.2006.103911 17138903 PMC1716240

[105] Viruell-Fuentes EA Miranda PY Abdulrahim S. More than culture: structural racism, intersectionality theory, and immigrant health. Soc Sci Med. (2012) 75:2099–106. 10.1016/j.socscimed.2011.12.037 22386617

[106] Idler EL Benyamini Y. Self-rated health and mortality: a review of twenty-seven community studies. J Health Soc Behav. (1997) 38:21–37. 10.2307/2955359 9097506

[107] Assari S Lapeyrouse LM Neighbors HW Income and self-rated mental health: diminished returns for high income black Americans. Behav Sci. (2018) 8:50 10.3390/bs8050050 PMC5981244 29772799

[108] Assari S Caldwell CH. Family income at birth and risk of attention deficit hyperactivity disorder at age 15: racial differences. Children. (2019) 6:10. 10.3390/children6010010 30646634 PMC6352113

[109] Assari S. The benefits of higher income in protecting against chronic medical conditions are smaller for African Americans than whites. Healthcare. (2018) 6:2. 10.3390/healthcare6010002 29315227 PMC5872209

[110] Assari S. Educational Attainment Better Protects African American Women than African American men against depressive symptoms and psychological distress. Brain Sci. (2018) 8:182. 10.3390/brainsci8100182 30274391 PMC6210325

[111] Assari S. High Income Protects Whites but Not African Americans against risk of depression. Healthcare. (2018) 6:37. 10.3390/healthcare6020037 29690595 PMC6023547

[112] Assari S Bazargan M. Educational attainment better reduces disability for non-hispanic than Hispanic Americans. Eur J Investigation Health Psychol Educ. (2019) 10:10–7. 10.3390/ejihpe10010002 32089977 PMC7034936

[113] Williams DR Mohammed SA. Discrimination and racial disparities in health: evidence and needed research. J Behav Med. (2009) 32:20–47. 10.1007/s10865-008-9185-0 19030981 PMC2821669

[114] Assari S Caldwell CH Zimmerman MA. Family structure and subsequent anxiety symptoms; minorities' diminished return. Brain Sci. (2018) 8:97. 10.3390/brainsci8060097 29857488 PMC6025006

[115] Assari S Mistry R Bazargan M. Race, educational attainment, and e-cigarette use. J Med Res Innovation. (2020) 4:e000185. 10.32892/jmri.185 32090188 PMC7034862

[116] Assari S Bazargan M. Unequal effects of educational attainment on workplace exposure to second-hand smoke by race and ethnicity; minorities' diminished returns in the National Health Interview Survey (NHIS). J Med Res Innov. (2019) 3:179. 10.32892/jmri.179 31404444 PMC6688774

[117] Assari S Mistry R. Diminished return of employment on ever smoking among hispanic whites in Los Angeles. Health Equity. (2019) 3:138–44. 10.1089/heq.2018.0070 31289772 PMC6608689

[118] Assari S BM. Second-hand exposure home second-hand smoke exposure at home in the United States; minorities' diminished returns. Int J Travel Med Glob Health. (2019) 7:28. 10.15171/ijtmgh.2019.28 32195278

[119] Assari S Lankarani MM. Education and alcohol consumption among older Americans; black-white differences. Front Public Health. (2016) 4:67. 10.3389/fpubh.2016.00067 27148514 PMC4838609

[120] Assari S Lankarani MM. Educational attainment promotes fruit and vegetable intake for whites but not blacks. J (Basel). (2018) 1:29–41. 10.3390/j1010005 31844842 PMC6914217

[121] Assari S Bazargan M. Minorities' diminished returns of educational attainment on hospitalization risk: National Health Interview Survey (NHIS). Hosp Pract Res. (2019) 4:86–91. 10.15171/hpr.2019.17 31650101 PMC6812545

[122] Markides KS Rote S Immigrant health paradox. Emerg Trends Soc Behav Sci. (2015) 2015:1–15. 10.1002/9781118900772.etrds0174

[123] Strompolis M Tucker W Crouch E Radcliff E. The intersectionality of adverse childhood experiences, race/ethnicity, and income: implications for policy. J Prev Interv Community. (2019) 47:310–24. 10.1080/10852352.2019.1617387 31131725

[124] Troe EJ Kunst AE Bos V Deerenberg IM Joung IM Mackenbach JP. The effect of age at immigration and generational status of the mother on infant mortality in ethnic minority populations in The Netherlands. Eur J Public Health. (2007) 17:134–8. 10.1093/eurpub/ckl108 16877451

[125] Weisman de Mamani A Weintraub MJ Maura J Martinez de Andino A Brown CA Gurak K. Acculturation styles and their associations with psychiatric symptoms and quality of life in ethnic minorities with schizophrenia. Psychiatry Res. (2017) 255:418–23. 10.1016/j.psychres.2017.06.074 28672225

[126] Manly JJ Byrd DA Touradji P Stern Y. Acculturation, reading level, and neuropsychological test performance among African American elders. Appl Neuropsychol. (2004) 11:37–46. 10.1207/s15324826an1101_5 15471745

[127] Otiniano Verissimo AD Gee GC Ford CL Iguchi MY. Racial discrimination, gender discrimination, and substance abuse among Latina/os nationwide. Cultur Divers Ethnic Minor Psychol. (2014) 20:43–51. 10.1037/a0034674 24491127 PMC4060885

[128] Borjas GJ Tienda M. The employment and wages of legalized immigrants. Int Migration Rev. (1993) 27:712–47. 10.1177/019791839302700401 12286923

[129] Kahn JR Whittington LA The labor supply of Latinas in the USA: comparing labor force participation, wages, and hours worked with Anglo and Black women. Population Res Policy Rev. (1996) 15:45–77. 10.1007/BF00156742

[130] Mahler SJ American Dreaming: Immigrant Life on the Margins. Princeton University Press (1995). Available online at: https://www.hofstra.edu/pdf/academics/colleges/hclas/cld/cld_rlr_s99_americandream.pdf (accessed October 10, 2020).

[131] Mendes MM Representations about discrimination practices in the education system built by russian and ukrainian immigrants'children in lisbon metropolitan. Trames. (2009) 13:341–56. 10.3176/tr.2009.4.02

[132] Tannock S Points of prejudice: education-based discrimination in Canada's immigration system. Antipode. (2011) 43:1330–56. 10.1111/j.1467-8330.2010.00864.x

[133] Steinmann JP The paradox of integration: why do higher educated new immigrants perceive more discrimination in Germany? J Ethnic Migration Stud. (2019) 45:1377–400. 10.1080/1369183X.2018.1480359

[134] Dietz J Introduction to the special issue on employment discrimination against immigrants. J Managerial Psychol. (2010) 25:104–12. 10.1108/02683941011019320

[135] Crane HM Rompaey SEV Dillingham PW Herman E Diehr P Kitahata MM. A single-item measure of health-related quality-of-life for HIV-infected patients in routine clinical care. AIDS Patient Care STDs. (2006) 20:161–74. 10.1089/apc.2006.20.161 16548713

[136] DeSalvo KB Fisher WP Tran K Bloser N Merrill W Peabody J. Assessing measurement properties of two single-item general health measures. Quality Life Res. (2006) 15:191–201. 10.1007/s11136-005-0887-2 16468076

[137] Van Hooff ML Geurts SA Kompier MA Taris TW. “How fatigued do you currently feel?” Convergent and discriminant validity of a single-item fatigue measure. J Occupational Health. (2007) 49:224–34. 10.1539/joh.49.224 17575403

[138] Kempen GI Miedema I van den Bos GA Ormel J. Relationship of domain-specific measures of health to perceived overall health among older subjects. J Clin Epidemiol. (1998) 51:11–8. 10.1016/S0895-4356(97)00234-5 9467630

[139] Youngblut JM Casper GR. Focus on psychometrics single-item indicators in nursing research. Res Nursing Health. (1993) 16:459–65. 10.1002/nur.4770160610 8248573 PMC3601191

[140] Ahmad F Jhajj AK Stewart DE Burghardt M Bierman AS. Single item measures of self-rated mental health: a scoping review. BMC Health Services Res. (2014) 14:398. 10.1186/1472-6963-14-398 25231576 PMC4177165

[141] Bize R Plotnikoff RC. The relationship between a short measure of health status and physical activity in a workplace population. Psychol Health Med. (2009) 14:53–61. 10.1080/13548500802032699 19085312

[142] de Boer AG van Lanschot JJ Stalmeier PF van Sandick JW Hulscher JBF de Haes JCJM. Is a single-item visual analogue scale as valid, reliable and responsive as multi-item scales in measuring quality of life? Quality Life Res. (2004) 13:311–20. 10.1023/B:QURE.0000018499.64574.1f 15085903

[143] DeSalvo KB Jones TM Peabody J McDonald J Fihn S Fan S. Health care expenditure prediction with a single item, self-rated health measure. Med Care. (2009) 47:440–7. 10.1097/MLR.0b013e318190b716 19238099

[144] Rohland BM Kruse GR Rohrer JE Validation of a single-item measure of burnout against the Maslach Burnout Inventory among physicians. Stress Health. (2004) 20:75–9. 10.1002/smi.1002

[145] Bulli F Miccinesi G Maruelli A Katz M Paci E. The measure of psychological distress in cancer patients: the use of distress thermometer in the oncological rehabilitation center of florence. Supportive Care Cancer. (2009) 17:771. 10.1007/s00520-008-0543-9 19050940

[146] Robins RW Hendin HM Trzesniewski KH Measuring global self-esteem: construct validation of a single-item measure and the rosenberg self-esteem scale. Personality Soc Psychol Bull. (2001) 27:151–61. 10.1177/0146167201272002

[147] Elo A-L Leppänen A Jahkola A. Validity of a single-item measure of stress symptoms. Scand J Work Environt health. (2003) 29:444–51. 10.5271/sjweh.752 14712852

[148] Patrick-Miller L Broccoli T Much J Levine E Validation of the distress thermometer: a single item screen to detect clinically significant psychological distress in ambulatory oncology patients. J Clin Oncol. (2004) 22(14_suppl):6024 10.1200/jco.2004.22.14_suppl.6024

[149] Cunny KA Perri M III. Single-item vs multiple-item measures of health-related quality of life. Psychol Reports. (1991) 69:127–30. 10.2466/pr0.1991.69.1.127 1961779

[150] Lefèvre T Singh-Manoux A Stringhini S Dugravot A Lemogne C Consoli SM. Usefulness of a single-item measure of depression to predict mortality: the GAZEL prospective cohort study. Eur J Public Health. (2012) 22:643–7. 10.1093/eurpub/ckr103 21840893 PMC3457003

[151] Assari S Lankarani MM Burgard S. Black-white difference in long-term predictive power of self-rated health on all-cause mortality in United States. Ann Epidemiol. (2016) 26:106–14. 10.1016/j.annepidem.2015.11.006 26803458 PMC4792698

[152] Assari S. Gender differences in the predictive role of self-rated health on short-term risk of mortality among older adults. SAGE Open Med. (2016) 4:2050312116666975. 10.1177/2050312116666975 27651902 PMC5019363

[153] Assari S. Psychiatric Disorders Differently Correlate with Physical Self-Rated Health across Ethnic Groups. J Pers Med. (2017) 7:6. 10.3390/jpm7040006 29137173 PMC5748622

[154] Cobb S Assari S. Self-Rated health in African American Men and Women; evidence for sponge hypothesis. Int J Environ Res Public Health. (2020). 7:25–34. 10.34172/ijer.2020.05 32395609 PMC7213594

